# *GABRG2 C588T* Polymorphism Is Associated with Idiopathic Generalized Epilepsy but Not with Antiepileptic Drug Resistance in Pakistani Cohort

**DOI:** 10.1155/2022/3460792

**Published:** 2022-11-15

**Authors:** Tayyaba Saleem, Hafsa Maqbool, Nadeem Sheikh, Asima Tayyeb, Maryam Mukhtar, Aqsa Ashfaq

**Affiliations:** ^1^Cell and Molecular Biology Laboratory, Institute of Zoology, University of the Punjab, Lahore, Pakistan; ^2^School of Biological Sciences, University of the Punjab, Lahore, Pakistan

## Abstract

Idiopathic generalized epilepsy (IGE) is the most prevalent type of epilepsy with genetic origin. Mutations in ion channel genes have been identified as a common cause of IGE. Several studies have reported various epilepsy risk variants of *GABRG2* (*gamma-aminobutyric acid type A receptor subunit gamma2 subunit*) gene in different ethnic groups, but the results are inconsistent. The purpose of this case-control research is to determine if *GABRG2* polymorphisms contribute to IGE susceptibility and antiepileptic drug resistance in Pakistani population. For this purpose, we genotyped exon2, exon5 (*C540T* and *C588T*), exon7 (*T813C*), exon8 (*K289M*), and exon9 of *GABRG2* gene by restriction fragment length polymorphism and Sanger's sequencing in 87 drug-responsive idiopathic generalized epilepsy patients, 55 drug-resistant epilepsy patients, and 83 healthy controls. Restriction fragment length polymorphism (RFLP) and sequencing results indicated only *C588T* polymorphism in the studied subjects. The comparison of genotypic and allelic frequencies showed significant differences between IGE patients and control groups (*P* = 0.008 and odds ratio = 4.2) and nonsignificant association of C588T polymorphism in antiseizure medication-resistant patients (*P* = 0.9). Our findings showed that *C588T* polymorphism of *GABRG2* is a risk variant for IGE in Pakistani population. Further studies are required to validate the results.

## 1. Introduction

Epilepsy is a clinically and genetically heterogeneous group of conditions marked by episode of prolonged synchronized neuronal activity [1]. It is a prevalent disorder with a global incident rate of 7 per 1000 individuals [2]. The interplay of genetic and environmental factors causes the majority of epilepsy manifestations [3]. Idiopathic generalized epilepsy is the most common category of epilepsy with nonfocal mechanism of onset and no external cause or no cause beyond genetic predisposition according to the current definition [4]. Patient experiences seizures that entail both hemispheres of the brain. Although lGEs have a high incidence rate, they are still underdiagnosed. IGE accounts for 30 percent of all epilepsies, and around 0.3% of the general population is affected by it [5]. Idiopathic generalized epilepsies are considered polygenic based on high concordance between monozygotic twins and decelerating risk beyond first-degree relatives [6–8].

Seizures, epileptogenesis, and epilepsy are all influenced by genes and their variants on numerous levels. Voltage-gated and ligand-gated are key ion channel genes that have been linked to distinct epilepsy phenotypes [9]. Among ligand-gated channel genes, the genes encoding gamma-aminobutyric acid (GABA) receptors are considered a hotspot for susceptibility of IGE because of the extensive distribution of GABA receptors in the central nervous system (CNS), their potential for postsynaptic inhibition, and regulation by therapeutically important antiepileptic drugs [10]. Recently, multiple mutations in gamma 2 subunit of GABA receptors are discovered in two families which furnished the genetic evidence for possible role of GABA receptor system in epileptogenesis. In one of these families, the phenotype was identified to be consistent with generalized epilepsy and febrile seizures. The affected members of the second family experienced febrile seizures in addition to childhood absence epilepsy [11, 12].

Gamma-aminobutyric acid (GABA) receptor is a ligand-gated chloride channel. In the central nervous system principle, GABA receptor includes three subunits, i.e., *α*1, *β*2, and *γ*2. Malfunction of the gene encoding these subunits influences the expression, gating of the ion channels, and trafficking of GABA receptors to the cellular surface [13]. *GABRG2*, a highly expressed gene in brain, resides on chromosome 5q34. Individuals having mutated *GABRG2* are prone to febrile seizures, childhood absent epilepsy, and generalized epilepsy with febrile seizures plus [14].

Multiple studies conducted around the globe have reported mutations in both the exonic and intronic regions of *GABRG2* to be associated with different epilepsy phenotypes. The silent (*C588T*) [15, 16], missense (*R82Q*, *R177G*, and *K328M*) [11, 12, 17], nonsense (*Q390X*, *Q40X*, and *W429X*) [15, 18], and intronic *IVS6+2TG* [15] are reported to alter the expression and composition of GABAA receptor subunits, affecting transcriptional and translational efficacy, sensitivity to extrinsic environmental signals, and altered current kinetics or impaired oligomerization, resulting in epilepsy in various ethnic groups [15].

Important pharmacological targets for the regulation of neuronal activity in the brain are considered influenced by mutations in ion channel genes. GABAA receptors are major targets for antiepileptic medicines such as benzodiazepines, phenobarbital, gabapentin, and topiramate. In a rat model of temporal lobe epilepsy, it was recently discovered that antiseizure medication- (ASM-) resistant rats differ from medication-responsive rats in GABAA receptor subunit expression. It also suggests that changes in GABAA receptor subunits may play a role in ASM resistance [19, 20].

A novel way to classify complex gene-associated disorders like IGE can be offered by single-nucleotide polymorphism markers. The current research was devised to investigate the relationship between genetic variation in *GABRG2* gene and predisposition to IGE and its association with antiepileptic drug resistance in Pakistani population.

## 2. Materials and Methods

### 2.1. Subjects

The current study included 88 patients suffering from idiopathic generalized epilepsy clinically diagnosed by neurophysicians, 84 normal healthy volunteers as control, and 55 drug-resistant epilepsy patients. All 88 IGE patients were drug-responsive. All participants signed a consent form before enrollment in the study. All participants were recruited from the same geographical location and ethnicity to avoid potential biasness. A detailed questionnaire was administered to collect information on demographic and clinical attributes. Patients were eligible if they had drug-resistant or drug-responsive epilepsy, as described by the International league against epilepsy (ILAE) criteria, and had been taking antiseizure medication (ASM) for at least a year. The patients were excluded if they have substantial psychiatric comorbidities, an uncertain record of seizure frequency, experienced pseudoseizures, received inconsistent ASM therapy, drug addiction, and/or occurrence of neurodegenerative disorders. Drug resistance was defined as consistent seizure frequency despite treatment with a maximum tolerated dose of two established ASM. Drug responsiveness was characterized as full freedom from seizures for at least a couple of years in epileptic patients treated with ASM. The current study was approved by the Bioethics Committee of Institute of Zoology, University of Punjab, Lahore, Pakistan.

### 2.2. Sampling and DNA Isolation

From each participant, 3 cc venous blood was collected in EDTA-coated tubes and stored at 4°C till further processing. The identification of subject information and genotype data was done using a code to achieve blind genotyping. The DNA was isolated by modified organic method [21] with a final concentration of 50 ng per microliter in diethyl pyrocarbonate water (DEPC, ROTH Art.-Nr. T143.3). The confirmation of DNA was done by running 2 *μ*l of DNA with 6x loading dye on a 2% agarose gel, visualized under UV transilluminator (GelDoc Bio Imaging System). The quantification of DNA was done using NanoDrop spectrophotometer (OptizanNanoQ). The purity of DNA was accessed at 260/280 nm. The isolated DNA was stored at -20°C.

### 2.3. Selection of *GABRG2* Regions and Primer Optimization

In current study, we targeted exon2, exon5 (C540T and C588T), exon7 (T813C), exon8 (K289M), and exon9 of *GABRG2* gene. Previously reported forward and reverse primers were used for exons 5, 7, and 8. The primers for exon 2 and 9 were specifically synthesized for this study using Primer 3 plus software. The forward and reverse primer sequences are given in Table 1.The primers were ordered from Macrogen. The primers were optimized by gradient polymerase chain reaction in a total reaction mixture of 12 *μ*l containing 1.5 *μ*l of DNA, 0.75 *μ*l of forward and reverse primers, 3 *μ*l of master mix (amaROnePCR; Cat. No. SM213-0250), and 6 *μ*l of nuclease free water. The optimized annealing temperature of the primers was 59°C for exon 2, 60.5°C for exon 5, 47°C for exon 7, 57°C for exon 8, and 59°C for exon 9. The amplicons of 415 bp (exon 2), 413 bp (exon 5), 332 bp (exon 7), 307 bp (exon 8), and 397 bp (exon 9) were resolved on 2% agarose gel electrophoresis.

### 2.4. Genotyping

#### 2.4.1. PCR-Based Restriction Fragment Length Polymorphism (PCR-RFPL)

For genotypic analysis, DNA was amplified by polymerase chain reaction in a total reaction mixture of 25 *μ*l containing 2 *μ*l of DNA, 1.5 *μ*l of forward and reverse primers, 9 *μ*l master mix, and 11 *μ*l of nuclease free water. The thermocycling conditions were as follows: one cycle of denaturation at 95°C for 5 min, followed by 30 cycles of denaturation at 95°C for 30 sec, annealing at optimized annealing temperature for each pair of primers (mentioned above) for 45 sec, extension at 72°C for 45 sec, and final cycle of extension at 72°C for 10 min. PCR products were digested with restriction enzymes according to the manufacturer's instructions. Briefly, for exon 5 (C588T), the amplified PCR products were digested with ApoI restriction enzyme in a total reaction mixture of 31 *μ*l containing 10 *μ*l of PCR, 18 *μ*l of nuclease free water, 2 *μ*l of 10x buffer tango, and 1 *μ*l of ApoI enzyme. Reaction mixture was incubated for 5 hours at 37°C followed by incubation for 20 min at 80°C for thermal inactivation of enzyme. For exon 5 (C540T), the restriction digestion was carried out in a 5-hour incubation period at 37°C in 31 *μ*l reaction mixture (containing 10 *μ*l of PCR, 18 *μ*l of nuclease free water, 2 *μ*l of 10x buffer tango, and 1 *μ*l of BsmI enzyme) followed by inactivation at 65°C for 20 min. For exon 8 (K289M), the restriction digestion was carried out in a total reaction mixture of 31 *μ*l containing 10 *μ*l of PCR, 18 *μ*l of nuclease free water, 2 *μ*l of 10x buffer tango, and 1 *μ*l of NcoI enzyme. The reaction mixture was incubated at 37°C for 5 hours and then inactivated by brief incubation at 65°C for 20 min. After digestion, 5 *μ*l of each digested sample was run on 2% agarose gel for 25 min and visualized under UV transilluminator.

#### 2.4.2. Direct Sequencing

After initial screening of mutations in exons 5 and 8 by RFLP, all samples for exons 2, 5, 7, 8, and 9 were sequenced from a commercial source. For sequencing, the PCR products were prepared in a total reaction mixture of 25 *μ*l and amplified in thermocycling conditions described above. Sequences were visualized using BioEdit software and analyzed by NCBI blast.

### 2.5. Statistical Analysis

The demographic attributes were presented as mean ± S.D. The chi-square goodness-of-fit test was used to check the Hardy-Weinberg equilibrium (HWE) for genotype frequency distribution. The differences in genotypic and allelic frequencies of cases and controls were analyzed by Fisher's exact test. The association was expressed in terms of odds ratio or risk estimates with 95% confidence intervals. The statistical analysis was performed using SHEsis (http://analysis.bio-x.cn/myAnalysis.php) online software and SPSS software. The significance level for the test was <0.01.

## 3. Results

**Figure 1:**
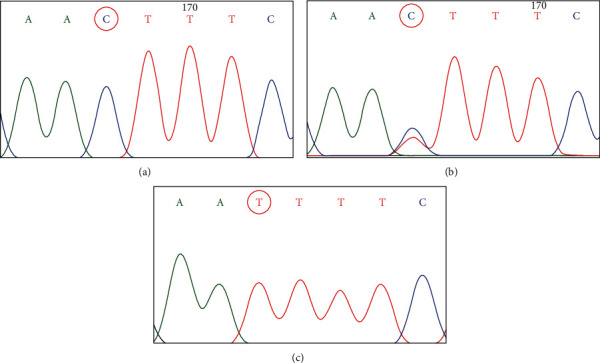
Electropherograms of exon 5 (C588T): (a) homozygous wild-type genotype CC, (b) heterozygous mutated genotype CT, and (c) homozygous mutated genotype TT.

The demographic and clinical variables of the case and control groups are summarized in Table 2. The sequence analysis showed only C588T polymorphism in both homozygous and heterozygous form (Figure 1). The substitution led to a silent change in amino acids. None of the other targeted polymorphisms were observed in the studied groups. The genotype proportions of the cases and controls fitted the Hardy-Weinberg equilibrium, as calculated by the *χ*^2^ test for polymorphism.

### 3.1. *GABRG2* (C588T) Association with IGE

**Figure 2:**
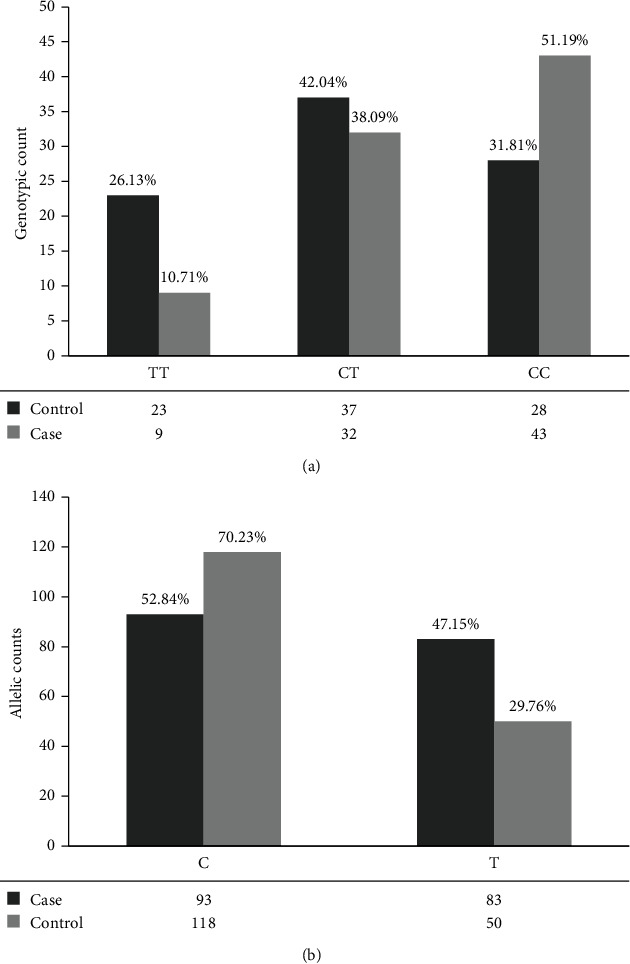
(a) Genotype frequency for *GABRG2* (C588T) in the case and control groups and (b) allelic frequencies for *GABRG2* (C588T) in the case and control groups.

The genotypic (CC, CT, and TT) counts and percentage for the case and control groups are presented in Figure 2. The test statistics showed a significant difference between CC, CT, and TT genotypic frequencies of the two groups (*P* < 0.01). T/C heterozygote was the most prevalent genotype for *GABRG2* (*rs211037*) gene in IGE patients, whereas C homozygote was the most common genotype in the control group. In individuals with idiopathic generalized epilepsies, the *GABRG2*- (*rs211037*-) TT genotype was overrepresented relative to healthy control participants (IGE = 26.13 percent vs. control = 10.71 percent, *P* = 0.008). When compared to the *GABRG2*- (*rs211037*-) CC genotype, the odds ratio for developing idiopathic generalized epilepsies in people with the *GABRG2*- (*rs211037*-) TT genotype was 4.2 (95%CI = 1.7 − 10.2). When compared to the *GABRG2*- (*rs211037*-) CC genotype, the odds ratio for developing idiopathic generalized epilepsies among those with the *GABRG2*- (SNP211037-) TT and *GABRG2*- (*rs211037*-) CT genotype was 1.7. Allelic frequencies in the case and control group are summarized in Figure 2. The C allele was taken as reference. Allelic frequencies differ significantly in the case and control groups with T allele significantly frequent in IGE patients (*P* = 0.0009) as shown in Table 3. The odds ratio for developing IGE in individuals with T allele was 2.15 (95% CI, 1.4-3.2).

### 3.2. *GABRG2* Association with Antiepileptic Drug Resistance

In the ASM-resistant group, the proportions of homozygous T, heterozygous CT, and homozygous CC genotypes for C588T were 7 (12.7%), 21(38.2%), and 27 (49.1%), respectively. The frequency of allele C and T was 75 (68.2%) and 35 (31.8%), respectively. The difference between the genotypic frequencies of the control group and ASM-resistant group was statistically insignificant (*P* = 0.9). The difference between allelic frequencies of these groups was not significant (*P* = 0.71, 95%CI = 0.53 − 1.52). Both groups have a higher percentage of C allele compared to T allele. The odds ratio for developing drug resistance in patients with T allele was 0.90. Similarly, when the ASM-resistant group was compared with the IGE group, the difference between genotypic frequencies of both groups was not significant (*P* = 0.05). The difference between genotypic and allelic frequencies was also not significant (*P* = 0.01, 95%CI = 1.16 − 3.14). The odds ratio for developing drug resistance in patients with T allele was 1.91.

## 4. Discussion

Previous research on the involvement of *GABRG2* in IGE has yielded conflicting results that may be attributed to ethnical differences. Therefore, the current study was undertaken to investigate the association of *GABRG2* polymorphism in exons 2, 5, 7, 8, and 9 with epilepsy susceptibility in Pakistani population. The results of our study showed that *rs211037* may be a risk variant for idiopathic generalized epilepsy in Pakistani cohort. GABAA receptor subunit gene mutations, particularly *GABRG2*, have been linked to the etiology of different kinds of epilepsy. Through pre- or posttranslational processes, these mutations affect GABAA receptor function and/or biogenesis [22]. The exonic *rs211037* polymorphism, interestingly, has no effect on the amino acid sequence (Asn196Asn) [23, 24]. Synonymous mutations have been linked to the likelihood of various complicated disorders in recent genomic research. In addition to its involvement in regulating protein posttranslational folding, the *rs211037* polymorphism has a crucial role in splicing and transcriptional organization, which requires more investigation [25].

We find a link between SNP- (*rs211037*-) TT genotype and IGE. Butilă et al. [26] found that among Romanian individuals with idiopathic generalized epilepsy, the mutant TT genotype increased up to 5.5 times higher than the CC and CT genotypes (*P* = 0.0009). When compared to well-controlled children (*P* = 0.06), Ponnala et al. [27] found a greater prevalence of the T allele in patients with generalized epilepsy (*P* = 0.05) and an increase in TT genotype carriers within the recurrent seizure group (*P* = 0.06). In contrast to our findings, a case-control study of 77 Taiwanese epileptic children and 83 control participants found a link between this variation and IGE. The frequency of the *GABRG2-* (*rs211037-*) C allele was substantially greater in patients than in healthy control people at*P* value of 0.002, according to the researchers. When comparing people with the CC genotype to those with the TT genotype, the OR for developing IGE was 3.61 [28].

*GABRG2* (*rs211037*) has a broad range of allele and genotype frequencies in different cultures throughout the world, indicating that ethnic differences may play a role in the distribution of this genetic variant. In fact, research on the genetic link between *rs211037* and febrile seizures (FS) backs up this hypothesis. Chou et al. studied 104 children and 83 control participants in a case-control study in the Taiwanese community. The C allele and the CC genotype are substantially more common in patients with FS, according to the researchers [29]. Similarly, it was hypothesized in a case-control research including 100 cases and 120 healthy controls that the same allele might be a good genetic marker for predicting susceptibility to FS in Egyptian children [14].

A study conducted in Indian population by Kumari et al. [3] showed no association between *GABRG2* (*rs211037*) and epilepsy susceptibility. In another study, genotype proportions and allele frequencies for the C558 T polymorphism were not different in 98 unrelated Brazilian individuals with juvenile myoclonic epilepsy (JME) [30]. Furthermore, there was no significant difference in genotype and allele distribution between German patients and controls with childhood idiopathic absence epilepsy (*P* = 0.35 and 0.49) [31].

GABA receptors are potential targets for antiepileptic therapy. Association studies on *GABRG2* polymorphism and antiepileptic drug response have conflicting results. The meta-analysis and in silico analysis conducted by Wang et al. [32] concluded that *GABRG2* might be a potential target for the treatment of epilepsy. We did not observe any association between *GABRG2* polymorphism and antiepileptic drug resistance. A study conducted in India on 441 subjects also reported a lack of the association between *GABRG2 C588T* polymorphism and ADR [33]. A study conducted on 401 Indian epileptic patients reported no association between *GABRG2 C588T* polymorphism and ADR [34]. In North India, a study conducted on 395 epilepsy patients showed no association between *GABRG2* polymorphism epilepsy and drug-refractive epilepsy [3]. In contrast, a case-control study conducted in Egyptian children showed a statistically significant association between *GARG2 C588T* substitution and drug resistance. Compared with drug responders, the authors reported an increase in T allele in the drug-resistant group (OR = 4.09, CI = 7.91 − 2.12, *P* = 0.00015) [35]. A higher frequency of T allele was also reported by Butilă et al. in Romanian patients (*P* = 0.001; OR = 5.29) [26]. The phenotypic variability (patient characteristics) and the flexible description of antiepileptic medication resistance may explain the discrepancies across research publications studying the connection between the GABAA receptor polymorphism and treatment resistance.

## 5. Limitations of the Study

The study's primary limitation is the small number of IGE patients in our cohort. We did not break them down into subcategories and do analysis for the whole group of IGE since there were not enough participants in each category to allow for an adequate statistical evaluation and findings.

## 6. Conclusion

This study suggested that the *GABRG2 C588T* polymorphism might pose a risk for idiopathic generalized epilepsy but not for ADR in Pakistani population. However, large-scale testing and functional characterization are required to confirm our findings. Our results will aid in developing population-specific markers. In the future, more association studies in global populations, combining diverse candidate genes and samples with carefully specified symptoms, might help researchers better understand the genetic predisposition to this epileptic disease.

## Figures and Tables

**Table 1:** The forward and reverse primer sequences of targeted exons.

	Forward primer	Reverse primer	Reference
Exon 2	CAGTTAGTCTCCATCTATGCAG	TCCTTGCTCTTGAACTACACTG	[11, 18]
Exon 5	CCTGGACTTGGTGGATTTCTTC	TCACCCTAATCGGAGCAAGCTG	
Exon 7	GCAGATCAACATAGAAAT	AATGTGTGTGCATAACC	
Exon 8	CACGAGTGACTCAGTTACCC	ATTTCAATGGTGCCAATGG	
Exon 9	GCTCAGAACTCTCCTTCTGTG	TAGCTTTTGGGCTTGGTGTAAG	

**Table 2:** Demographic and clinical attributes of the participants.

Variable	IGEs group 88 (%)	Control group 84 (%)	ASM-resistant group 55 (%)
Age in years (mean ± SD)	13 ± 2.21	12 ± 2.59	7 ± 1.5
Age at onset in years			
Gender			
Male	47 (53.4090)	45 (53.57)	31 (56.36)
Female	41 (46.59)	39 (46.42)	24 (43.63)
Family history			
First-degree relatives	17 (19.31)	—	11 (20)
Second-degree relatives	9 (10.22)	—	21 (38.18)
Seizure type			
Myoclonic jerks	17 (19.31)	—	8 (14.5)
Generalized tonic clonic seizures	32 (36.36)	—	29 (52.72)
Absence seizures	27 (30.68)	—	19 (34.54)
Tonic	9 (10.22)	—	9 (16.36)
Juvenile absence seizures	15 (17.045)	—	7 (12.72)
Treatment			
Monotherapy	35 (39.77)	—	6 (10.90)
Polytherapy	53 (60.22)	—	49 (89.09)

**Table 3:** Genotypic and allelic frequencies in numbers (*N*).

	IGE group (*N*)	Control group (*N*)	Drug resistant	
*Genotype*				
TT	23	9	7	
CT	37	32	21	
CC	28	43	27	
*Allele*				
C	93	118	75	
T	83	50	35	

## Data Availability

The data used to support the findings of this study is available upon request from the corresponding author.
